# Perceived Obstacles of Colorectal Cancer Screening and Their Associated Factors among 10,078 Chinese Participants

**DOI:** 10.1371/journal.pone.0070209

**Published:** 2013-07-23

**Authors:** Martin C. S. Wong, Jessica Y. L. Ching, Hoyee H. Hirai, Thomas Y. T. Lam, Sian M. Griffiths, Francis K. L. Chan, Joseph J. Y. Sung

**Affiliations:** 1 Institute of Digestive Disease, Faculty of Medicine, Chinese University of Hong Kong, Hong Kong SAR, China; 2 School of Public Health and Primary Care, Chinese University of Hong Kong, Hong Kong SAR, China; University of Missouri Kansas CIty School of Medicine, United States of America

## Abstract

**Purpose:**

to evaluate the proportion of self-referred screening participants having various psychological barriers and the factors associated with these barriers.

**Methods:**

A territory-wide bowel cancer screening centre sent an invitation via the media to all Hong Kong residents aged 50–70 years who were asymptomatic of CRC to join a free screening programme. Upon attendance they were requested to complete self-administered surveys on their perceived barriers of screening. Binary logistic regression analyses were used to evaluate the factors associated with these barriers.

**Results:**

From 10,078 consecutive screening participants (mean age 57.5 years; female 56.4%) completed the surveys between May 2008 to September 2012. There were high proportions who agreed or strongly agreed with the following barriers: financial difficulty (86.0%), limited service accessibility (58.2%), screening-induced bodily discomfort (55.2%), physical harm (44.4%), embarrassment (40.1%), apprehension (38.8%) and time constraints (13.9%). From regression models, older participants (aged ≥56) were less likely to have these barriers (Adjusted odds ratio [AOR] ranged from 0.738 to 0.952) but they encountered more difficulties to access to screening services (AOR ranged from 1.141 to 1.371). Female subjects were more likely to encounter most of these barriers (AOR ranged from 1.188 to 2.179). Participants who were uncertain of the necessity of CRC screening for people aged ≥50 were more likely to report these barriers (AOR ranged from 1.151 to 1.671).

**Conclusion:**

The proportions of perceptual barriers of CRC screening were high among these participants. Those with these associated factors should receive more thorough explanation of the screening test procedures.

## Introduction

Worldwide, colorectal cancer (CRC) is the second and third most common malignancy in women and men, respectively. It accounts for 10% of all cancers globally, leading to 8% of all cancer mortalities in the world, and is the fourth commonest cause of cancer death [1]. In the past decades, Asia-Pacific countries such as China, South Korea, Japan and Singapore have witnessed a two to three-fold rise in incidence of CRC [2], and have been gradually catching up with the figures in Western countries like the US and the UK. The direct medical cost for the care of colorectal neoplasia was estimated to be US$1,941 for low risk polyps and US$45,115 for stage IV CRC in the initial year of care, leading to a substantial global public health burden to the healthcare systems [3].

Three landmark randomised controlled trials have shown that CRC screening using Faecal Occult Blood Testing (FOBT) is effective in reducing cancer mortality by 15% to 33% [4]–[6]. A 25% relative risk reduction in CRC mortality was found for those attending at least one round of FOBT screening, according to a systematic review conducted in 2007 [7]. Screening colonoscopy studies have demonstrated that 0.5–1% of patients will have CRC diagnosed, and 5–10% of patients will have advanced neoplasia detected [8]. Guidelines from the US Preventive Services Task Force (USPSTF), the European Nations, the Asia-Pacific Consensus statements and other authorities [9]–[12] recommended average risk individuals aged 50 to 70 years to undergo CRC screening, which are compatible with more recent guidelines from the American Cancer Society, Multi-Society Task Force on CRC and the American College of Radiology [12]–[14].

However, studies from Japan, France, England and Hong Kong showed that participation or compliance rates in CRC screening using FOBT remained low, ranging from 10–61% [15]–[18]. A recent territory-wide population-based survey conducted in Hong Kong showed that perceived access, health and psychological barriers to CRC screening were strongly associated with lower screening uptake rates [18]. Prominent psychological barriers of CRC screening included concerns about pain, discomfort, unpleasantness associated with CRC testing and fear of follow-up procedures. Lack of insurance coverage is also a significant barrier, as there is an absence of incentive to undertake screening when no symptoms exist. Financial cost and lack of time commitment are the major access barriers to the undertake CRC screening tests [18]. Other studies have reported that embarrassment associated with CRC screening has been a particularly important obstacle to undergo a screening test [19]–[21].

Nevertheless, most of the existing studies on barriers to CRC screening were conducted among the general public, family members of CRC patients and those who declined invitations to screening programmes. In primary care settings, subjects who expressed an initial interest to CRC screening are arguably the most likely group who will eventually receive a CRC screening test, but there exists no studies conducted among these individuals. The objective of this study is to evaluate the proportion of self-referred CRC screening participants who perceived various psychological barriers, and the independent factors associated with perception of these barriers.

## Materials and Methods

### Ethics Statement

This study was approved by the Clinical Research Ethics Committee of the Chinese University of Hong Kong.

### Setting

A CRC screening centre was established in Hong Kong in 2008, which invited free CRC screening via the media for all Hong Kong residents aged 50–70 years asymptomatic of CRC. A more detailed description of this invitation has been published previously [22], [23]. Briefly, this study was conducted in a community-based centre which provides education and CRC screening to a large population of Hong Kong. Data were collected based on recruitment between 1^st^ May 2008 and 31^st^ July 2012.

### Study Design

This study prospectively recruited a consecutive cohort of 10,078 participants aged 50 to 70 years who self-referred for CRC screening in the centre via telephone, fax, email, or walk-in.

### Participant Recruitment

The eligibility criteria for this study was (i) age 50 to 70 years; (ii) absence of existing or previous symptoms suggestive of CRC such as haematochezia, malena, anorexia or change in bowel habit in the past 4 weeks, or weight loss greater than 5 kg in the past 6 months; and (iii) absence of screening test for CRC performed in the past 5 years. Exclusion criteria included personal history of CRC, colonic adenoma, diverticular disease, inflammatory bowel disease, prosthetic heart valve or vascular graft surgery. Participants with medical conditions which were contraindications for colonoscopy were also excluded [22]. The eligibility of each participant and the exclusion criteria were checked by trained staff.

Registered participants were invited to fill in a self-administered questionnaire. Meanwhile, centre staff checked for the completeness of questionnaires and trained volunteers assisted survey completion for illiterate participants by reading the questions word-by-word. Information on their age, gender, educational level, marital status, occupation, monthly household income, family history of CRC was collected. They were also enquired on their perception of various perceptual barriers to CRC screening. A four-point Likert scale was adopted to assess perceptions of eight barriers to CRC screening (strongly agree; agree; disagree; strongly disagree), developed based on published methodology using the Health Belief Model [22], [24], [25], and validated by a panel of epidemiologists, psychologists and gastroenterologists.

### Outcome Variables and Covariates

The outcome variables include the proportions of screening participants who agreed or strongly agreed the presence of eight perceived barriers. These included screening-induced physical harm, bodily discomfort, embarrassment, apprehension, financial difficulties, time constraints for attending screening programmes, limited accessibility to screening service providers, and perceived benefits of screening being minimal. The covariates tested for association with these barriers include participants’ age, gender, educational level, marital status, occupational status, monthly household income, self-perceived risks of CRC, family history, and the necessity of CRC screening for people aged ≥50 years.

### Statistical Analyses

All data were entered into a predesigned database with logistic checking using Microsoft Access, and analyzed using SPSS software, version 16.0 (Chicago, Illinois). The proportions of participants who perceived the barriers were compared according to the covariates. Eight separate, unconditional logistic regression analyses were conducted with all covariates listed above entered into the models after checking for the absence of interactions. The adjusted Odds Ratios (AORs) and 95% CIs of the potential independent predictors of perceived barriers were estimated. All the variables selected in the multivariate regression analysis were detected for the presence of co-linearity (r >0.80) [26]. P-values ≤0.05 were regarded as statistically significant.

## Results

### Participant Characteristics

A total of 10,078 screening participants were included in the analysis (**Table 1**). Their mean age was 57.5 years (SD 5.12 years), and 56.4% were female. The majority of them (56.9%) achieved an educational level at secondary or above, and were married or cohabiting (84.5%). 35.8% worked full-time and 34.0% worked in part-time jobs or were retired. Most of them (57.4%) had monthly household income at US$2,571 or below, and 68.2% perceived themselves as at risks for developing CRC. 13.1% and 12.3% reported their first and second degree relatives to have suffered from CRC, respectively. 83.4% of the participants regarded CRC screening for people aged 50 years or older as very or quite necessary.

**Table 1:** Participant Characteristics (N = 10,078).

	No. of Participants	Proportions
***Age (years)***		
**50–54**	3408	33.8
**55–59**	3244	32.2
**60–64**	2280	22.6
**65–70**	1136	11.3
***Gender***		
**Male**	4384	43.5
**Female**	5689	56.4
***Educational level***		
**Primary or below**	2747	27.3
**Secondary**	5739	56.9
**Tertiary or above**	1576	15.6
***Marital status***		
**Married/cohabit**	8514	84.5
**Single/divorced/widowed/others**	1546	15.3
***Occupational status***		
**Full time**	3609	35.8
**Part time or retired**	3424	34.0
**Housewife and others**	3030	30.1
***Monthly household income ($US)***		
**<1285$**	2932	29.1
**1285$ – 2571$**	2856	28.3
**2571$ – 3856$**	1428	14.2
**3856$ – 5141$**	665	6.6
**>5142$**	611	6.1
**Refused to answer**	1572	15.6
***Self perceived risk of CRC***		
**At risk**	6873	38.2
**Not at risk**	2552	25.3
**Not sure**	608	6.0
***Family history of CRC***		
**Nil**	5714	57.7
**First degree relatives**	1313	13.0
**Second degree relatives**	1242	12.3
**Others**	1709	17.0
***Necessity of CRC screening for people aged ≥50***		
**Very or quite necessary**	8402	83.4
**Not very necessary or unnecessary**	344	3.4
**Not sure**	1315	13.0

a CRC: Colorectal Cancer.

### Levels of Perceived Barriers among Screening Participants

Financial difficulty (86.0%), limited service accessibility (58.2%) and screening-induced bodily discomfort (55.2%) were the barriers where the greatest proportions of participants “agreed” or “strongly agreed” as barriers; these were followed by physical harm (44.4%), embarrassment (40.1%), apprehension (38.8%) and time constraints (13.9%) (**Table 2**). A minority perceived the benefit of CRC screening was minimal (9.5%).

**Table 2:** Attitudinal Barriers of colorectal cancer (CRC) screening (N = 10,078).

	Physical harm		Bodilydiscomfort		Embarrassment		Apprehension		Economic difficulties		Time constraint		Poor accessibility		Perceived benefit being minimal	
	n^a^	%^a^	n	%	n	%	n	%	n	%	n	%	n	%	n	%
***Age (years)***																
**50–54**	1512	44.4	1898	55.7	1512	44.4	1373	40.3	2951	86.6	522	15.3	2008	58.9	329	9.7
**55–59**	1473	45.4	1805	55.6	1351	41.6	1274	39.3	2762	85.1	482	14.9	2067	63.7	310	9.6
**60–64**	1020	44.7	1288	56.5	833	36.5	858	37.6	1973	86.5	277	12.1	1534	67.3	208	9.1
**65–70**	466	41.0	570	50.2	338	29.8	351	30.9	976	85.9	122	10.7	790	69.5	109	9.6
***Gender***																
**Male**	1784	40.7	2151	49.1	1390	31.7	1224	27.9	3668	83.7	625	14.3	2716	62.0	380	8.7
**Female**	2690	47.3	3414	60.0	2648	46.5	2686	47.2	4998	87.9	779	13.7	3688	64.8	576	10.1
***Educational level***																
**Primary or below**	1304	47.5	1696	61.7	1204	43.8	1245	45.3	2427	88.4	477	17.4	2034	74.0	346	12.6
**Secondary**	2497	43.5	3043	53.0	2245	39.1	2101	36.6	5034	87.7	720	12.5	3512	61.2	499	8.7
**Tertiary or above**	670	42.5	823	52.2	585	37.1	560	35.5	1205	76.5	206	13.1	853	54.1	110	7.0
***Marital status***																
**Married/cohabit**	3804	44.7	4657	54.7	3343	39.3	3264	38.3	7316	85.9	1172	13.8	5396	63.4	788	9.3
**Single/divorced/widowed/others**	665	43.0	902	58.3	690	44.6	643	41.6	1347	87.1	230	14.9	1001	64.7	166	10.7
***Occupational status***																
**Full time**	1516	42.0	1895	52.5	1393	38.6	1267	35.1	3014	83.5	760	21.1	2212	61.3	342	9.5
**Part time or retired**	1458	42.6	1827	53.4	1225	35.8	1182	34.5	2961	86.5	311	9.1	2183	63.8	300	8.8
**Housewife and others**	1498	49.4	1842	60.8	1418	46.8	1461	48.2	2690	88.8	332	11.0	2005	66.2	312	10.3
***Monthly household income ($US)***																
**<1285$**	1270	43.3	1641	56.0	1121	38.2	1120	38.2	2685	91.6	389	13.3	2008	68.5	288	9.8
**1285$ – 2571$**	1261	44.2	1600	56.0	1110	38.9	1065	37.3	2573	90.1	423	14.8	1838	64.4	271	9.5
**2571$ – 3856$**	619	43.3	731	51.2	549	38.4	500	35.0	1193	83.5	206	14.4	834	58.4	110	7.7
**3856$ – 5141$**	283	42.6	331	49.8	256	38.5	235	35.3	505	75.9	79	11.9	370	55.6	51	7.7
**>5142$**	259	42.4	293	48.0	251	41.1	238	39.0	363	59.4	92	15.1	296	48.4	49	8.0
**Refused to answer**	781	49.7	969	61.6	749	47.6	752	47.8	1348	85.8	214	13.6	1053	67.0	186	11.8
***Self perceived risk of CRC***																
**At risk**	3095	45.0	38.9	40.5	2788	40.6	2654	38.6	5944	86.5	342	5.0	4330	63.0	611	8.9
**Not at risk**	1103	43.2	1390	54.5	996	39.0	994	38.9	2167	84.9	300	11.8	1616	63.3	261	10.2
**Not sure**	261	42.9	342	56.3	237	39.0	250	41.1	524	86.2	312	51.3	431	70.9	79	13.0
***Family history of CRC***																
**Nil**	2497	43.7	3178	55.6	2347	41.1	2282	39.9	4992	87.4	886	15.5	3872	67.8	608	10.6
**First degree relatives**	590	44.9	722	55.0	532	40.5	495	37.7	1140	86.8	176	13.4	761	58.0	97	7.4
**Second degree relatives**	567	45.7	714	57.5	483	38.9	470	37.8	1085	87.4	137	11.0	754	60.7	105	8.5
**Others**	821	48.0	953	55.8	678	39.7	666	39.0	1454	85.1	205	12.0	1018	59.6	146	8.5
***Necessity of CRC screening for people aged ≥50***																
**Very or quite necessary**	3682	43.8	4541	54.0	3301	39.3	3110	37.0	7249	86.3	1068	12.7	5172	61.6	707	8.4
**Not very necessary or unnecessary**	169	49.1	217	63.1	149	43.3	163	47.4	291	84.6	68	19.8	230	66.9	67	19.5
**Not sure**	620	47.1	803	61.1	586	44.6	637	48.4	1124	85.5	267	20.3	995	75.7	182	13.8

a All n/% refer to responses of “strongly agree” or “agree”.

### Factors Associated with the Perception of Screening Barriers

From multivariate regression analysis, older age was significantly associated with lower likelihood of perceiving screening-induced bodily discomfort, embarrassment, apprehension, and financial difficulties (**Table 3** **and** **Table 4**). Nevertheless, they were more likely than younger participants to have poorer access to service providers. Female participants were more likely to encounter perception of physical harm, bodily discomfort, embarrassment and apprehension related to the screening process, as well as time constraints to attend screening sessions (Adjusted odds ratios [AOR] ranged from 1.188 to 2.179). In general, participants with higher educational levels were less likely to encounter all of these barriers separately (AOR ranged from 0.531 to 0.894). Marital status was not associated with perception of any barriers. When compared with subjects with full-time jobs, those with part-time jobs, retired or housewives were less likely to encounter embarrassment (AOR 0.854 to 0.865), perceive time constraints (AOR 0.317 to 0.325) and experience accessibility problems (AOR 0.861 to 0.876). In addition, housewives were more likely to perceive physical harm induced by screening (AOR 1.217). Participants having monthly household income >US$5,142 were more likely to feel embarrassed (AOR 1.285) and apprehensive (AOR 1.385) about screening, yet were in general less likely to encounter financial difficulties (AOR 0.137 to 0.782), time constraints (AOR 0.661 to 0.816), and accessibility to screening services (AOR 0.580 to 0.710). Those who did not perceived themselves at risks for CRC were less likely to experience physical harm (APR 0.897), embarrassment (AOR 0.884), apprehension (AOR 0.920) and financial difficulties (AR 0.853). Participants with their relatives having family history of CRC were less likely to encounter accessibility problems (AOR 0.729 to 0.799). Except financial difficulties, people who were uncertain about the necessity of CRC screening among subjects aged at ≥50 years were more likely to encounter all the barriers under study (AOR 1.151 to 1.671). The covariates in the regression analysis did not show interactions nor multi-collinearity, implying the robustness of the regression models.

**Table 3:** Factors associated with greater barriers of CRC screening (physical harm, bodily discomfort, embarrassment and apprehension).

	Physical harm		Bodily discomfort		Embarrassment		Apprehension	
	Adjusted odds ratio (95% C.I.)	p	Adjusted odds ratio (95% C.I.)	p	Adjusted odds ratio (95% C.I.)	p	Adjusted odds ratio (95% C.I.)	p
*Age (years)*								
50–54	1.00 (referent)		1.00 (referent)		1.00 (referent)		1.00 (referent)	
55–59	1.016 (0.919–1.123)	0.754	0.949 (0.858–1.049)	0.305	0.879 (0.794–0.972)	0.012	0.952 (0.858–1.056)	0.352
60–64	0.982 (0.874–1.105)	0.766	0.966 (0.858–1.087)	0.561	0.702 (0.622–0.791)	<0.001	0.883 (0.782–0.998)	0.046
65–70	0.866 (0.744–1.008)	0.063	0.738 (0.634–0.859)	<0.001	0.520 (0.443–0.611)	<0.001	0.830 (0.709–0.972)	0.021
*Gender*								
Male	1.00 (referent)		1.00 (referent)		1.00 (referent)		1.00 (referent)	
Female	1.188 (1.076–1.311)	0.001	1.411 (1.278–1.558)	<0.001	1.708 (1.543–1.891)	<0.001	2.179 (1.965–2.417)	<0.001
*Educational level*								
Primary or below	1.00 (referent)		1.00 (referent)		1.00 (referent)		1.00 (referent)	
Secondary	0.894 (0.812–0.985)	0.024	0.761 (0.690–0.840)	<0.001	0.865 (0.783–0.955)	0.004	0.812 (0.735–0.898)	<0.001
Tertiary or above	0.905 (0.786–1.042)	0.166	0.835 (0.725–0.963)	0.013	0.854 (0.738–0.988)	0.034	0.880 (0.759–1.020)	0.089
*Marital status*								
Married/cohabited	1.00 (referent)		1.00 (referent)		1.00 (referent)		1.00 (referent)	
Single/divorced/widowed/others	0.892 (0.795–1.001)	0.051	1.008 (0.897–1.132)	0.893	1.095 (0.975–1.230)	0.126	0.930 (0.826–1.047)	0.228
*Occupational status*								
Full time	1.00 (referent)		1.00 (referent)		1.00 (referent)		1.00 (referent)	
Part time or retired	1.052 (0.944–1.173)	0.360	1.009 (0.905–1.125)	0.871	0.865 (0.783–0.955)	0.004	0.975 (0.869–1.093)	0.659
Housewife and others	1.217 (1.078–1.374)	0.001	1.058 (0.936–1.196)	0.365	0.854 (0.738–0.988)	0.034	1.103 (0.975–1.248)	0.121
*Monthly household income ($US)*								
<1285$	1.00 (referent)		1.00 (referent)		1.00 (referent)		1.00 (referent)	
1285$ – 2571$	1.061 (0.951–1.183)	0.292	1.041 (0.932–1.162)	0.475	1.024 (0.914–1.146)	0.685	1.050 (0.937–1.177)	0.400
2571$ – 3856$	1.055 (0.922–1.208)	0.435	0.880 (0.769–1.007)	0.064	1.025 (0.891–1.178)	0.733	0.986 (0.856–1.137)	0.850
3856$ – 5141$	1.052 (0.879–1.259)	0.580	0.863 (0.722–1.033)	0.108	1.104 (0.917–1.328)	0.295	1.071 (0.887–1.293)	0.478
>5142$	1.078 (0.886–1.311)	0.455	0.827 (0.680–1.005)	0.056	1.285 (1.052–1.569)	0.014	1.385 (1.131–1.696)	0.002
*Self perceived risk of CRC*								
At risk	1.00 (referent)		1.00 (referent)		1.00 (referent)		1.00 (referent)	
Not at risk	0.897 (0.817–0.985)	0.023	0.917 (0.835–1.007)	0.069	0.884 (0.803–0.972)	0.011	0.920 (0.835–1.013)	0.091
Not sure	0.878 (0.739–1.042)	0.136	0.950 (0.799–1.129)	0.557	0.904 (0.757–1.078)	0.260	0.976 (0.817–1.165)	0.784
*Family history of CRC*								
Nil	1.00 (referent)		1.00 (referent)		1.00 (referent)		1.00 (referent)	
First degree relatives	1.079 (0.954–1.220)	0.224	1.010 (0.893–1.142)	0.875	0.975 (0.860–1.106)	0.696	0.943 (0.829–1.072)	0.368
Second degree relatives	1.101 (0.972–1.247)	0.131	1.103 (0.973–1.252)	0.127	0.901 (0.792–1.025)	0.114	0.895 (0.785–1.020)	0.096
Others	1.236 (1.108–1.379)	<0.001	1.066 (0.954–1.191)	0.257	0.956 (0.854–1.071)	0.437	0.984 (0.877–1.103)	0.778
*Necessity of CRC screening for people aged ≥50*								
Very or quite necessary	1.00 (referent)		1.00 (referent)		1.00 (referent)		1.00 (referent)	
Not very necessary or unnecessary	1.198 (0.962–1.492)	0.106	1.332 (1.061–1.673)	0.014	1.145 (0.915–1.432)	0.238	1.402 (1.121–1.754)	0.003
Not sure	1.151 (1.020–1.298)	0.022	1.283 (1.134–1.451)	<0.001	1.250 (1.106–1.413)	<0.001	1.571 (1.389–1.777)	<0.001

a CRC: Colorectal Cancer.

* Figures in red bold represent the association was tested significant (p<0.05).

**Table 4:** Factors associated with greater barriers of CRC screening (economic difficulties, time constraints, poor accessibility, perceived benefit of screening being minimal).

	Economic difficulties		Time constraint		Poor accessibility		Perceived benefit being minimal		
	Adjusted odds ratio(95% C.I.)	p	Adjusted odds ratio(95% C.I.)	p	Adjusted odds ratio(95% C.I.)	p	Adjusted odds ratio(95% C.I.)	p	
***Age (years)***									
**50–54**	1.00 (referent)		1.00 (referent)		1.00 (referent)		1.00 (referent)		
**55–59**	0.738 (0.635–0.856)	<0.001	1.094 (0.950–1.260)	0.213	1.141 (1.029–1.266)	0.012	0.953 (0.805–1.129)	0.579	
**60–64**	0.738 (0.617–0.883)	0.001	1.051 (0.883–1.251)	0.576	1.263 (1.117–1.429)	<0.001	0.857 (0.701–1.047)	0.130	
**65–70**	0.660 (0.526–0.828)	<0.001	1.039 (0.820–1.318)	0.749	1.371 (1.166–1.611)	<0.001	0.885 (0.685–1.142)	0.347	
***Gender***									
**Male**	1.00 (referent)		1.00 (referent)		1.00 (referent)		1.00 (referent)		
**Female**	1.116 (0.963–1.292)	0.144	1.206 (1.048–1.388)	0.009	1.021 (0.921–1.132)	0.696	1.063 (0.897–1.259)	0.481	
***Educational level***									
**Primary or below**	1.00 (referent)		1.00 (referent)		1.00 (referent)		1.00 (referent)		
**Secondary**	1.064 (0.913–1.240)	0.429	0.638 (0.556–0.732)	<0.001	0.634 (0.570–0.705)	<0.001	0.730 (0.625–0.853)	<0.001	
**Tertiary or above**	0.749 (0.614–0.913)	0.004	0.668 (0.543–0.821)	<0.001	0.531 (0.458–0.616)	<0.001	0.586 (0.455–0.755)	<0.001	
***Marital status***									
**Married/cohabited**	1.00 (referent)		1.00 (referent)		1.00 (referent)		1.00 (referent)		
**Single/divorced/widowed/others**	0.873 (0.733–1.039)	0.126	1.066 (0.904–1.257)	0.446	0.977 (0.866–1.103)	0.712	1.130 (0.937–1.362)	0.203	
***Occupational status***									
**Full time**	1.00 (referent)		1.00 (referent)		1.00 (referent)		1.00 (referent)		
**Part time or retired**	1.007 (0.859–1.181)	0.932	0.317 (0.269–0.374)	<0.001	0.861 (0.768–0.964)	0.009	0.867 (0.719–1.046)	0.137	
**Housewife and others**	1.050 (0.872–1.265)	0.606	0.325 (0.273–0.387)	<0.001	0.876 (0.771–0.995)	0.041	0.888 (0.725–1.087)	0.248	
***Monthly household income ($US)***									
**<1285$**	1.00 (referent)		1.00 (referent)		1.00 (referent)		1.00 (referent)		
**1285$–2571$**	0.782 (0.648–0.944)	0.010	0.903 (0.769–1.061)	0.216	0.900 (0.802–1.011)	0.076	1.008 (0.838–1.213)	0.933	
**2571$–3856$**	0.433 (0.353–0.532)	<0.001	0.816 (0.669–0.996)	0.046	0.720 (0.626–0.829)	<0.001	0.831 (0.652–1.058)	0.133	
**3856$–5141$**	0.267 (0.211–0.338)	<0.001	0.661 (0.502–0.872)	0.003	0.689 (0.574–0.828)	<0.001	0.882 (0.637–1.221)	0.450	
**>5142$**	0.137 (0.108–0.174)	<0.001	0.828 (0.625–1.097)	0.188	0.580 (0.476–0.707)	<0.001	1.021 (0.720–1.448)	0.906	
***Self perceived risk of CRC***									
**At risk**	1.00 (referent)		1.00 (referent)		1.00 (referent)		1.00 (referent)		
**Not at risk**	0.853 (0.745–0.977)	0.021	0.956 (0.834–1.097)	0.523	0.958 (0.869–1.056)	0.386	1.089 (0.932–1.272)	0.283	
**Not sure**	0.931 (0.723–1.199)	0.580	0.905 (0.708–1.156)	0.424	1.120 (0.927–1.353)	0.239	1.249 (0.964–1.619)	0.092	
***Family history of CRC***									
**Nil**	1.00 (referent)		1.00 (referent)		1.00 (referent)		1.00 (referent)		
**First degree relatives**	1.032 (0.858–1.243)	0.735	0.880 (0.735–1.054)	0.165	0.729 (0.643–0.827)	<0.001	0.740 (0.590–0.928)	0.009	
**Second degree relatives**	1.075 (0.888–1.301)	0.457	0.727 (0.597–0.884)	0.001	0.799 (0.702–0.909)	0.001	0.831 (0.667–1.035)	0.098	
**Others**	0.956 (0.814–1.122)	0.578	0.783 (0.662–0.925)	0.004	0.781 (0.697–0.876)	<0.001	0.845 (0.698–1.024)	0.086	
***Necessity of CRC screening for people aged ≥50***									
**Very or quite necessary**	1.00 (referent)		1.00 (referent)		1.00 (referent)		1.00 (referent)		
**Not very necessary or unnecessary**	0.718 (0.527–0.979)	0.036	1.644 (1.239–2.182)	0.001	1.057 (0.836–1.337)	0.642	2.288 (1.723–3.039)	<0.001	
**Not sure**	0.850 (0.712–1.014)	0.071	1.671 (1.428–1.955)	<0.001	1.661 (1.446–1.906)	<0.001	1.532 (1.278–1.835)	<0.001	

a CRC: Colorectal Cancer.

* Figures in red bold represent the association was tested significant (p<0.05).

## Discussion

At present CRC screening among asymptomatic patients in Hong Kong is not subsidized by the Government and citizens who wish to undergo screening should pay out of their own pocket. According to the Health Belief Model [27] one should address the major constructs in order to enhance the CRC screening uptake rate. These include perceived susceptibility, severity, barriers and benefits. In this study we found a high proportion of CRC screening participants having various barriers, especially financial difficulty, limited service accessibility, as well as screening-induced bodily discomfort, physical harm, embarrassment and apprehension. Notable patient groups having higher likelihood of encountering these barriers include younger subjects, female participants, people with lower educational level, subjects with full-time jobs, those who perceived themselves at risks for CRC, and people who were uncertain about the necessity of CRC screening among subjects aged at ≥50 years.

A cross-sectional study of the barriers among a sample of persons at risk for CRC was conducted in the Mid-western metropolitan area of Omaha [28]. A significant proportion of people reported internal barriers like time constraints (49%), pain (44%), inconvenience (42%), fear of cancer diagnosis (42%) and embarrassment (35%), amongst others. Some external factors were also reported, including cost of the screening tests (44%) and lack of recommendation from a primary care physician (35%). This study presented even higher proportions of screening participants reporting these barriers. Further, a recent systematic review includes 83 studies on the most commonly found barriers to CRC screening and the associated factors. Some of them were compatible with the findings of the present survey. These consist of low education levels, female gender, low socioeconomic status, presence of chronic comorbid conditions, being married or living with partner, lack of awareness regarding CRC screening, absence of health insurance and lack of screening recommendation by a physician [29]. The last barrier has been consistently found from other studies [30], [31], and also in our previous study [18]. However, we are unaware of any studies conducted among self-referred screening participants studying the independent factors associated with perception of the different barriers. The reasons why people with these associated factors were more likely to perceive screening barriers remain speculative, and future studies are warranted to ascertain the underlying reasons.

To our knowledge, this is the largest study conducted among screening participants on their perceptions of screening barriers. The survey used was designed based on the well recognized health belief model, and the 100% completion rates of the questionnaires are amongst some of the strengths. Nevertheless, there are some limitations which should be addressed. Firstly, the study was conducted among consecutive screening participants, and their socio-demographic characteristics might be different from the general public. However, as our research question focused on self-referred screening participants, it is inevitable for this survey to include more health-conscious subjects who are not generalisable to the population. In addition, some participants might have already accessed to information on the CRC screening tests before attendance to the centre, and this might change their perception on the different barriers. Furthermore, there might exist some confounders where we could not control for in this study, like prior experience of health service utilization, peer influences and previous consultations with physicians. Furthermore, this is a cross sectional study which could not delineate cause-and-effect relationships - and one may only draw conclusions on associations between the barriers and the covariates. Some of the other well-recognized barriers have not been evaluated, including those related to healthcare providers and the Governmental policy.

This study bears several important implications. Firstly, the proportions of participants having various barriers of CRC screening were high despite the fact that they were self-referred. It could be speculated the general public may experience these barriers to an even greater extent. To improve CRC screening uptake, more educational seminars should be designed and implemented in the community and clinics to explain the screening procedures in a more thorough manner. These include the simplicity and safety nature of the screening process which very rarely induces significant bodily discomfort or physical harm. Peer educators who have undergone CRC screening procedures could be invited to share with prospective screening participants on their screening experience, which could potentially remove the perception of embarrassment and apprehension associated with screening. Secondly, this study has evaluated the factors associated with the perception of these barriers. It follows that people with these associated factors should be explored more for the possible presence of psychological barriers. Also, more comprehensive explanation of the screening procedures should be discussed with this group of prospective participants to facilitate screening uptake.

In addition, it has been found that some health system-related barriers were also reported in a large proportion of participants. For instance, whereas the elderly were less likely to encounter the various psychological barriers and were at higher risks for CRC, they were however more likely to experience poor access to the service providers for screening. Also, those who perceived themselves at risks for CRC were more likely to experience psychological barriers, which might be due to their higher likelihood to encounter an adverse screening outcome. For implementation of population-based CRC screening programmes in the future, a better infrastructure for CRC screening should be constructed so as to improve accessibility to screening services, coupled with counseling services which could help remove the various barriers. Financial difficulties have also been reported in a large proportion of self-referred participants, and the Government should consider subsidizing screening services among eligible subjects as recommended by guidelines. In the long term, this will translate into reduction of CRC mortality. Future studies should explore effective interventional strategies to overcome these barriers.
